# Ectopic Scrotum with VACTERL Association

**DOI:** 10.21699/jns.v6i2.570

**Published:** 2017-04-15

**Authors:** Samiul Hasan, Ashrarur Rahman Mitul, Sabbir Karim

**Affiliations:** Department of Pediatric Surgery, Dhaka Shishu (Children) Hospital, Dhaka.

**Keywords:** Ectopic scrotum, VACTERL.

## Abstract

Scrotal ectopia is a rare condition. Associated anomalies are common. We describe a neonate with ectopic scrotum with VACTERL association. This combination of anomalies is very rare.

## Case report

**Figure 1:**
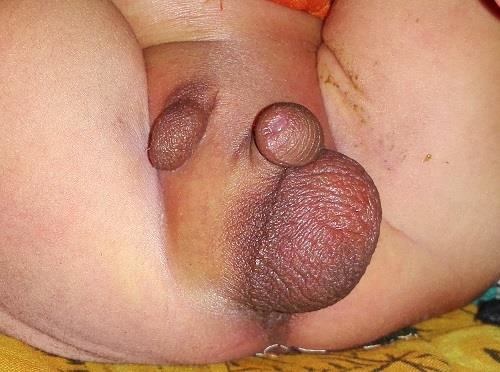
Ectopic scrotum.

A 1-day-old male neonate presented with ectopic scrotum and anorectal malformation (ARM). Clinically revealed, ARM with urinary fistula, right hemiscrotum present in right inguinal region, small in size and empty (Fig.1). Right testis was found bellow the superficial inguinal ring. Left hemiscrotum was normal in size and location with normal testis within. Scrotal raphe was normal and phallus was normally developed. This patient also had lumber scoliosis and bilateral talipes equinovarus. X-ray spine revealed lumber hemi vertebrae and scoliosis. On ultrasonography, right kidney found in left side of pelvis. Echocardiography showed patent foramen ovale and tricuspid regurgitation. Divided sigmoid colostomy done under general anaesthesia for ARM with urinary fistula. Excision of ectopic small right hemiscrotum and implantation of right testis in left hemiscrotum is planned at a later stage.

## Discussion 

It is believed that defect in the gubernacular development leads to ectopic scrotum [1,2]. Stephens proposed mechanical pressure on developing fetus responsible for this anomaly, which also explains the associated anomalies like anorectal malformation and TEV [2]. Both were present in our case. Ectopic scrotum mainly occurs in four locations – inguinal, supra-inguinal, infra-inguinal and perineal [3]. In this case this occurred in right inguinal region. Common associated anomalies include inguinal hernia, cryptorchidism and exstrophy bladder. About 70% of suprainguinal ectopic scrotums are associated with ipsilateral renal anomaly [3]. In our patient, ectopic kidney was found. VACTERL and VATER association with ectopic scrotum has been described by Spears T and Bawa M *et al*. [2,4]. Our patient also had vertebral, anorectal, cardiac, renal and limb anomaly. Ectopic scrotum without congenital anomalies are also reported [3]. Associated anomalies are common and all measures should be taken to exclude other anomalies including VACTERL association in all cases of ectopic scrotum.

## Footnotes

**Source of Support:** None

**Conflict of Interest:** None
